# How to Treat Peridontoclasia

**Published:** 1920-08

**Authors:** C. M. Gearhart


### HOW TO TREAT PERIDONTOCLASIA

BY C. M. GEARHART, D.D.S.

Barring apical infections, where, generally speaking, do you place the limitations to roots savable by periodontia? (a) For single-rooted teeth? (b) For lower molars? (c) For upper molars?

I would say that if fully one-half of the root is fixed in the alveolar process, and the patient's recuperative ability is good, an attempt to reclaim a tooth would be well justified. The same general idea would apply not only to single-rooted teeth, but to the multi-rooted teeth. There are exceptional cases in molar teeth, when we may find one root wholly denuded, where an amputation may be indicated, and the life of the tooth prolonged.

Do you believe a vital or well-filled pulpless root more favorable to periodontal regeneration?

In my opinion, a tooth with a healthy pulp has, without doubt, more favorable chances for periodontal regeneration. If the roots of the devitalized tooth, however, have been properly treated and filled, excellent results have been noted many times. If the devitalized tooth has ever been what Black described as "pus soaked," it should be immediately extracted.

What radiographic and clinical conditions will suggest testing the vitality of roots? (a) What is your technique for such tests?

There is no positive radiographic or clinical condition that is infallible in testing for the vitality of roots. Rarefied areas do not always appear coincidently with the death of the pulp. When we do find rarefied areas about the apices, however, we may be sure that the pulp is non-vital. (a) To test the vitality of a tooth, a lowered translucency may be noted by refraction. The Faradic battery is valuable also, in making this test. How many of us, though, have felt chagrined to learn after making these several tests that the tooth was still in a healthy and lively state?

What radiographic showing, independently of pus pockets, will indicate extracting roots and curetting sockets? (a) Do you advocate that procedure in the absence of apical rarefication? (b) Why?

Rarefication at the apices. (a) No, not unless metastatic or septicemic symptoms indicate systemic danger. (b) Because diploetic tissue yields to pericemental tumefaction, and because blood serum and leucocytes, plus high resistance, disposes of mild infection, and dissipates it in time, if it's cause is corrected.

Do you approve the temporary splinting of one or a few loose roots? (a) Under what circumstances? (b) If so, before, or immediately following curetting? (c) What kind of splint and how made? (d) How long should the splint be retained?

My experience with splints has been accompanied with great sorrow. Only in the most extreme cases and for some unusually good reason, can I conscientiously condone even temporary splints. I firmly believe that teeth, whose mobility is so great that they need be bolstered in position, should be removed without further thought.

What office prophylactic treatment would you advocate, if any, following curetting? (a) Its object? (b) Its technique? (c) How often repeated?

In answering these questions, I refer to my favorite definition for oral prophylaxis, *i. e.*, "Oral prophylaxis is a surgical interference for the prevention of diseases." It is, therefore, necessary only to preserve surgical cleanliness, and nature will do the rest.

